# Design of Aerated Oleogel–Hydrogel Mixtures for 3D Printing of Personalized Cannabis Edibles

**DOI:** 10.3390/gels10100654

**Published:** 2024-10-13

**Authors:** Eleftherios G. Andriotis, Adamantini Paraskevopoulou, Dimitrios G. Fatouros, Hui Zhang, Christos Ritzoulis

**Affiliations:** 1 Department of Food Science and Technology, International Hellenic University, 57400 Thessaloniki, Greece; 2 Laboratory of Food Chemistry and Technology, School of Chemistry, Aristotle University of Thessaloniki, 54124 Thessaloniki, Greece; 3 Laboratory of Pharmaceutical Technology, Department of Pharmacy, Faculty of Health Sciences, Aristotle University of Thessaloniki, 54124 Thessaloniki, Greece; 4 College of Biosystems Engineering and Food Science, Zhejiang University, Hangzhou 310058, China

**Keywords:** oleogels, bigels, cannabis seed oil, 3D printing, personalized dosage forms

## Abstract

Cannabis seed oil oleogel structured with Glycerol Monostearate (20% *w*/*w*) was mixed with xanthan gum hydrogel (2% *w*/*w*) at different ratios ranging from 0% *w*/*w* hydrogel to 75% *w*/*w* hydrogel, using a syringe-to-syringe apparatus, for the preparation of 3D-printable food inks. This process enabled the simultaneous blend of oleogel and hydrogel phases and the incorporation of air in a reproducible and accurate manner. The printability of bigel inks with different mass ratios was evaluated by using a conventional benchtop food 3D printer. The printability of the inks was found to be negatively affected by the presence of higher portions of the hydrogel phase, while the printing performance of pure cannabis seed oil oleogel was superior compared to the printing performance of the bigel inks. The physicochemical properties of hybrid gels were investigated with rheological studies, thermophysical studies (Differential Scanning Calorimetry), Polarized Light Microscopy, and Confocal Laser Scanning Microscopy. The microstructure of the aerated inks was affected by the presence of a higher oleogel fraction, in terms of air bubble shape and distribution. The addition of hydrogel at concentrations higher than 50% *w*/*w* had a strong negative effect on the mechanical properties of the inks leading to a partial collapse of the printed structures and subsequently to poor printing performance.

## 1. Introduction

*Cannabis Sativa* L. is one of the most recognizable and the oldest plants used for medical purposes, as it is known to contain a vast number of pharmaceutical compounds. There are more than 1200 different chemical compounds in the cannabis plant, while there are more than 100 known cannabinoids [1]. The hemp plant is also known for production of other products, like hemp or cannabis seed oil (CSO). CSO is very valuable in nutritional terms due to its composition (unsaturated fatty acids), and it is considered one of the best carriers for hosting active cannabinoids, like cannabidiol (CBD) or cannabigerol (CBG) [2]. Hempseeds are commonly consumed due to their pleasant nutty flavor and rich nutritional content [3]. They serve as an excellent source of essential fatty acids, minerals, vitamins, dietary fibers, and proteins such as edestin and albumin [3]. On the other hand, hempseed oil consists of polyunsaturated fatty acids, which are recognized for their positive impact on cardiovascular health, cancer prevention, and inflammatory conditions [3,4]. Consuming small quantities of hempseed oil (2 g/day) did not impact plasma levels of total cholesterol, high-density cholesterol (HDL), low-density cholesterol (LDL), or triglycerides. Additionally, it did not influence platelet aggregation or circulating inflammatory markers. While the evidence is limited, these findings imply that incorporating hempseed oil into one’s diet may offer potential health benefits [3,5]. On the other hand, the presence of polyunsaturated fatty acids in cannabis seed oil make it prone to oxidation during storage and exposure to heat. As a result, it is not recommended for food preparations that involve prolonged high-temperature cooking. To mitigate this issue, consumers can use smaller containers to manage consumption and ensure freshness [5]. The oxidative stability of cannabis seed oil is one of the reasons why this oil is not widely applied for the preparation of cannabinoids infused oil, suitable for the preparation of food products containing cannabis extract, known as cannabis edibles [6]. The concentration of active cannabinoids in edibles can vary across a single product and across batches formulated at different times, making it difficult for users to estimate the consumption amount. The lack of consistency may cause users of cannabis edibles to consume higher or lower than intended amounts of the active ingredients. Additive manufacturing of food systems can also be applied to address those issues. Food 3D printing (F3DP) is an emerging technology in the food research field, as it can provide novel routes for preparation of the food materials towards the fabrication of accurate 3D structures with defined textural properties [7,8].

The most widely applied method of food 3D printing is extrusion-based printing, which involves the extrusion of the food-stuff through a nozzle in the form of a paste [8,9]. Food-grade biopolymers such as xanthan, gellan gums, agar, carrageenan, alginate, gelatine, collagen, and chitosan are extensively studied as potential substrates for extrusion-based food 3D printing [10]. The final properties of the 3D printed specimen are highly dependent on the initial 3D printable ink properties, and subsequently through this dependent accuracy, personalization can be realized [11]. At this moment, the most extensively studied class of materials as 3D printable inks turns out to be hydrogels. Even though hydrogels can cover a wide range of structural and textural properties, with applications ranging from food products to pharmaceutical products for controlled drug release [12,13], they also have some limitations mainly in terms of printing precision and overall structural stability [8,14,15,16]. One suggested way to overcome these limitations is introducing solid lipids in the form of an oleogel, which could inhibit the network structure and alter the viscosity of the system, making it more stable [8,17]. Additionally, one of the most advantageous features of lipid-based food 3D printing is the broadening of the nutrient sources and the possibility of introducing poorly soluble substances to the system.

Bigels are biphasic systems consisting of phases with varying polarities. These systems exhibit stable structures characterized as viscoelastic semisolids [8,18,19], owing to the advantages of both hydrogel and organogel [8,20]. Additionally, bigels can be applied for the delivery of both hydrophilic and hydrophobic actives. They are easy to prepare and can be easily modified by simply adjusting the proportion of each phase in the system [8]. According to the existing literature, the application of bigels in 3D printing technology was justified by the fact that bigels could combine two phases and, subsequently, they could be a carrier system for more nutritional or pharmaceutical agents to enhance nutritional diversity [8] or personalized dosage forms for the accurate dosing of active pharmaceutical ingredients [1,21]. The 3D printing of such systems is already demonstrated in the literature where beeswax oleogel mixtures with carrageenan and xanthan gum [8] or candelilla wax oleogel mixed with gelatin hydrogel [22] were successfully printed using a 3D printer.

To ensure personalization and to avoid cannabis seed oil storage, processes that could be applied to small volumes of oil should be adopted. One such process is the syringe coupler method, inspired by the modified Tessari method for producing sclerosing foam [23]. This method is suitable for mixing small volumes (≤5 mL) and can be applied under fully aseptic conditions and even in the presence of inert gases like nitrogen [23]. The necessary equipment for the syringe coupler method is considered as low cost and easily accessible [23]. The method exploits the shear forces that are developed during compression and expansion of a mixture through a syringe nozzle [23]. The application of this method is always followed by the incorporation of a small amount of gas into the mixture, which is very difficult to avoid [23]. Nevertheless, the syringe coupler method can be applied for the precise incorporation of air into the system, towards the formation of foams with predetermined gas concentration [23].

To the best of our knowledge, there is a limited number of studies concerning the storage stability of cannabis seed oil, while there is no available data about the use of cannabis seed oil as a raw material for the preparation of 3D-printed cannabis edibles. In this study, cannabis edibles were considered as both food and dosage forms for the administration of cannabinoids. This approximation required the preparation of novel food systems in such a way that would be ready to host accurate doses of the active ingredients of cannabis. For this purpose, the technology of food additive manufacturing (food 3D printing) was applied to prepare precise and personalized cannabis edibles. To achieve this goal, 3D-printable food inks must be prepared with enhanced printability, to be suitable to serve as carriers for both hydrophilic and hydrophobic substances. The present study was focused on the development and evaluation of 3D-printable food systems that could be used as platform formulations (food printing inks) for printing food specimens with predetermined geometrical and physical characteristics. Thus, different formulations were characterized and evaluated for their printing performance by printing different model shapes like 1D, 2D, and 3D shapes.

## 2. Results and Discussion

### 2.1. Inks Appearance and Overrun Evaluation

**Figure 1:**
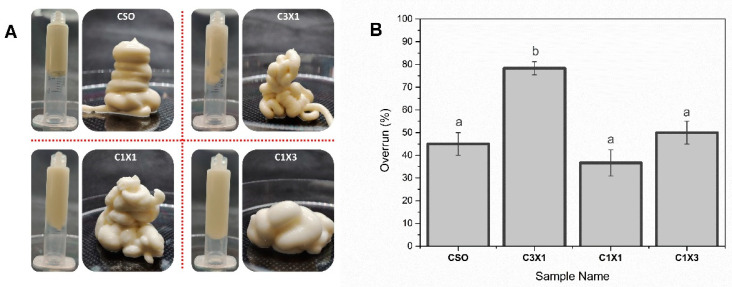
(**A**) Appearance evaluation and (**B**) overrun of the different ink formulations. Different lowercase letters indicate statistically significant differences.

The visual appearance of different formulations after the air-mixing process are exhibited in Figure 1A. In all cases, thick foam-like inks with excellent self-supporting characteristics were achieved. Additionally, the maximum overrun values for the different formulations are depicted in Figure 1B. All formulations were off-white, self-supporting semi-solid soft materials. The increase in the hydrogel portion in the formulation had a negative effect on the shape and uniformity of the foams. On the other hand, apart from the C3X1 sample, the maximum overrun was process-dependent, as there was no significant difference between the different samples. The C3X1 sample exhibited a higher overrun, suggesting a synergistic effect of the hydrogel on the stabilization of the air bubbles when used in smaller concentrations. The overall foaming properties of bigels with a higher concentration of oleogel (fatty acid crystals) agreed with the existing literature where it was reported that the foamability of bigels was affected by the presence of oleogel indicating that fatty acid crystals in the oil stabilized the air bubbles in whipped oleogels [24]. Conclusively, the as-prepared aerated bigels were considered visually homogeneous and were suitable for extrusion-based printing.

### 2.2. Printability Evaluation

**Figure 2:**
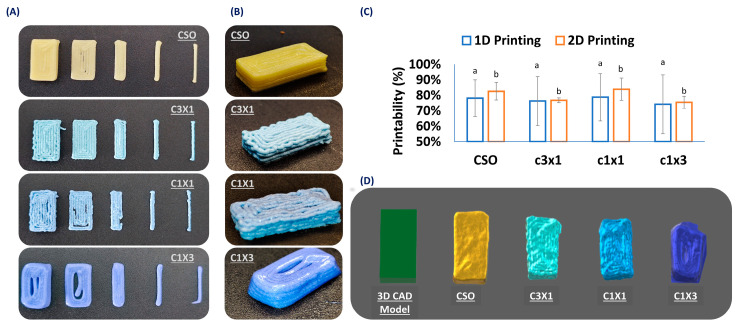
(**A**) Printability test of different formulations during 1D (single line), 2D (single layer), and 3D printing. (**B**) Printing performance of the different formulations (printing of a 3D rectangular shape; 10 mm × 20 mm × 5 mm). (**C**) Quantification of the printing performance for 1D and 2D printing. (**D**) 3D scanned printed specimens and comparison with initial 3D CAD model. Nile blue was used as a water-soluble colorant for the hydrogel phase. Different lowercase letters indicate statistically significant differences.

This study aimed to develop a 3D-printable food ink designed for the accurate dosing of cannabinoids. To ensure that the printed dosage form could be accurately printed, the printing process must be evaluated during the one-dimensional printing step (single-line filament extrusion), the two-dimensional printing step (single-layer printing), and finally the three-dimensional printing step (3D-printed specimen). The printing of the different model shapes are presented in Figure 2A. Specifically, the different printing steps that were in the printing process are separately discussed (namely 1D printing, 2D printing (2 mm, 3 mm, and 10 mm × 20 mm single layer), and 3D printing, from right to left, respectively). According to Figure 2A, all the different inks can be accurately printed in one-dimension, with minor defects, as in the case of C1X3, where there was minor material dragging by the nozzle. Nevertheless, the accuracy of the shape was followed by a discrepancy in the accuracy of the dimensions, compared to the given design, which stands for all the formulations. The deviation from the initial dimensions based on layer area is summarized in Figure 2C. When it came to the printing of a single layer, samples of CSO and C3X1 performed more accurately when compared to samples of C1X1 and C1X3. More specifically, in the case of the C1X1 formulation, there were structural failures due to extrusion discontinuity caused by partial nozzle clogging that could momentarily change the nozzle diameter (under-extrusion conditions). The partial clogging of the nozzle is attributed to possible inhomogeneity of the ink due to the manual mixing process. Total blockage of the nozzle was not observed as the printing was continued without over-extrusion signs. In the case of the C1X3 formulation, the under-extrusion conditions were more intense, resulting in a very round-shaped printing that deviated from the sharp edges of the initial design. The three-dimensional printing performance of the different formulations was evaluated by qualitatively comparing the 3D printed specimens (Figure 2B,D). As previously mentioned, in the case of samples of C1X1 and C1X3, there was a build-up of discontinuities caused by the continuous under-extrusion conditions. Additionally, C1X3 showed a partial layer collapse and failed to achieve self-support. The lack of self-support resulted in the specimen exhibiting a trapezoid shape instead of the intended rectangular shape of the initial design. The same deviation due to partial layer collapse was also observed for the samples of C3X1 and C1X1 but in a different way. Specifically, due to the differences in layer heights, the distance of the nozzle from the last layer was higher than 1 mm, causing the extruded material to drop from a distance. This behavior is directly linked to the rheological properties of the ink formulations, and it is furthered discussed in the respective section. These conditions caused the material to form curls that resulted in a less sharp shape. Finally, the printability of the CSO was the most optimum in terms of sharpness, shape accuracy, and homogeneity. It must be noted that the printing conditions could be adjusted to fit every formulation property and achieve a satisfactory printing performance.

### 2.3. Rheological Studies

The three pillars of extrusion-based three-dimensional printing could be described as (i) extrusion, (ii) recovery, and (iii) self-support. These distinctive pillars were directly related to the rheological properties of the extruded material and could be described by the viscosity, thixotropic characteristics, elastic modulus (G′), viscous modulus (G″), and complex modulus (G*) [8,25].

**Figure 3:**
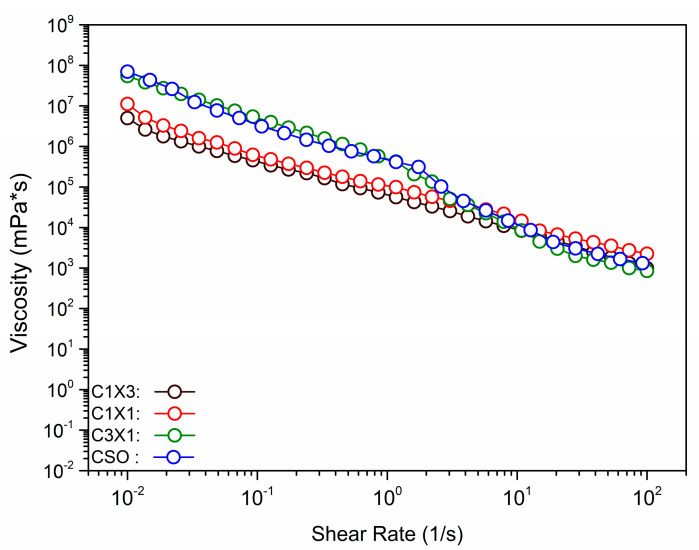
Viscosity vs. shear rate plot for the different ink formulations.

**Figure 4:**
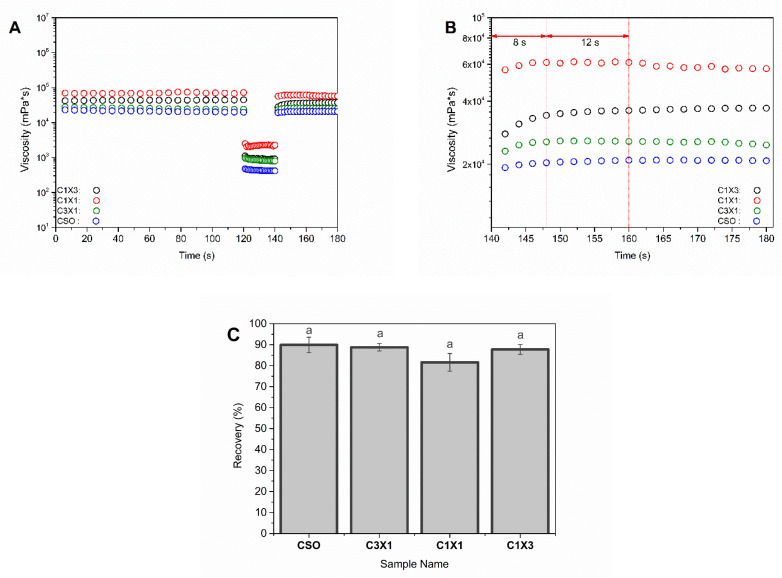
Viscosity recovery test for different ink formulations. (**A**) Three intervals thixotropy test, (**B**) recovery time, and (**C**) recovery percentage (the same letters indicate no statistically significant difference).

In Figure 3, the viscosity of the different formulations was plotted against the shear rate, to evaluate the rheological properties of the materials during the extrusion process. It was observed that the increase in shear rate was followed by a decrease in the apparent viscosity, for all samples, indicating that the inks were non-Newtonian fluids. Shear thinning is essential for 3D-printable inks to be able to be extruded through the print head nozzle smoothly. Both C1X1 and C1X3 samples had similar apparent viscosity values that were lower than those of the samples CSO and C3X1 at lower shear rates. On the other hand, CSO and C3X1 samples also possessed similar apparent viscosity values (between them) that were higher than those of the other two samples. For higher values of shear rates, all samples displayed similar apparent viscosity values. This phenomenon was also observed in previously reported studies for bigels when the fraction of hydrogel was higher than 50% *w*/*w*, indicating a system transition from a hydrogel-dominated system to an oleogel-dominated system [8]. All previous data were fitted using the Ostwald-de Waele power law model (Table 1). The power law index *n* could be used to evaluate the extrusion ability of the samples through the nozzle. For all samples, *n* values were less than 1, indicating that the inks exhibited a shear-thinning character. The negative *n* values of samples CSO and C3X1 indicate stronger shear-thinning properties. The power law index values were also divided into two groups, as was mentioned earlier. The shear thinning properties of all samples directly affect the extrudability and printability of the inks. Materials that exhibit shear-thickening properties induce over-extrusion conditions during printing, attributed to pressure built-up within the syringe barrel caused by nozzle clogging. The *K* value was indicative of the different apparent viscosity values of the samples. When aerated oleogel (CSO) was used, the *K* value was the maximum (Table 1), though without significant statistical difference from the next sample, which also had a higher oleogel fraction. These two samples differed significantly from the C1X1 sample which had almost half of the *K* value. Finally, the significantly lowest *K* value was observed for the sample C1X3, indicating that when the hydrogel fraction exceeded 50%, there was a decrease in viscosity, due to the domination of the hydrogel phase caused by hydrophobic interactions [8,25,26,27,28,29]. The higher *K* values indicated that the system showed solid-like characteristics during extrusion, meaning that the risk of clotting is higher [25]. As was previously discussed, there were no clogging signs (instantaneous over-extrusion) during the printing of all the under-study materials. This analysis supported the already discussed printability of the systems, where the printing of the CSO sample resulted in the sharpest and most accurate printed shape, and it can be correlated to the samples’ higher *K* and lower *n* values. Following the extrusion process during printing, the inks must recover their initial solid-like state (recovery). The ability of the inks to recover was evaluated by the application of the three intervals thixotropy test (Figure 4A).

All samples were subjected to the thixotropy test and exhibited a similar ability to recover their initial viscosity up to 89.93% (CSO sample, Figure 4C) following the termination of the high-speed shearing rate. The recovery time of the samples was defined as the time required for the samples to reach a plateau. In all cases, the viscosity reached a plateau within the first 8 s, and the viscosity was kept constant, indicating that the samples can self-support after the extrusion through the nozzle. It should be noted that samples of C1X1 and C3X1 showed a mild decrease in the viscosity after 20 s. This decrease could be explained by the partial collapse of the printed layers resulting to the formation of the curls and coils during printing (Figure 4B). This discrepancy could be considered as a longer recovery time for these samples and was consistent with the existing literature where it was reported that the recovery time of the printed gels was directly related to the ability of the inks to maintain their structure and their mechanical properties after 3D printing. Even though oleogels were known to lack strong connection points between crystals [8,30], in the present study, there was an opposite effect. Both CSO and C1X3 had a very fast recovery time, while the samples with intermediate concentrations (C3X1 and C1X1) exhibited a decrease in viscosity after the first 20 s, indicating that the recovery time was (in this case) oleogel-fraction-independent.

**Figure 5:**
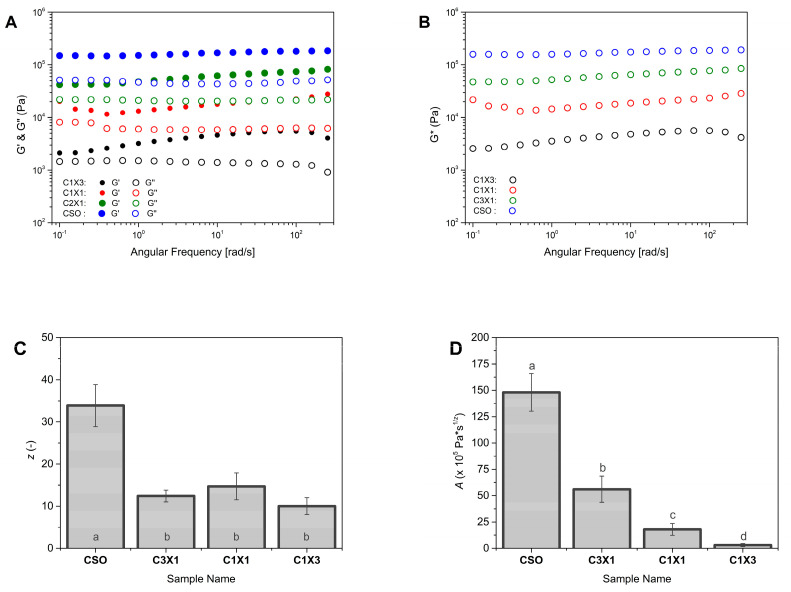
Rheological properties of the different ink formulations. (**A**) Elastic modulus (G′) and viscous modulus (G″) vs. frequency plots, (**B**) complex modulus (G*) vs. frequency plots, and weak gel model parameter in terms of (**C**) network strength; (**A**,**D**) network strength of network extension (z). Different lowercase letters indicate statistically significant differences.

The elastic modulus (G′), viscous modulus (G″), and complex modulus (G*) could be used to study the mechanical properties of the samples and provide us with information concerning their ability to self-support after 3D printing [8,31]. Figure 5A showed that the G′ of all samples was higher than the G″, indicating that the elastic properties of the inks were stronger than plastic properties (liquid-like properties) [8]. Additionally, the G* values increased as the oleogel fraction increased (Figure 5B), meaning that the inks with higher oleogel fractions tended to be mechanically stronger. This observation agreed with the printability evaluation that was previously discussed where specimens printed with inks with higher oleogel fractions performed in a better way. The smallest amount of oleogel fraction used (sample C1X3) was the least stiff among the samples that were studied. This difference in the stiffness was considered the main reason for the partial layer collapse that caused the formation of a trapezoid-shaped specimen instead of a rectangular shape, as is presented in Figure 2B, which was attributed to partial collapse of the printed layers. According to the weak gel model, the increased values of z indicated an increase in the interaction numbers of the bigel inks [8]. On the other hand, the same model dictated that the increased values of A were an indication of stronger interactions within the gel network [8,28]. Figure 5C revealed that the number of interactions (z values) for all samples were similar, except for CSO samples where the z value was considerably higher. The higher z value of CSO sample is directly linked with the better printing performance of this ink (Figure 2B). Figure 5D reveals a stronger dependency of the oleogel fraction on the overall strength of interactions within the gel network, with the CSO sample owning a considerably higher value of A. For the sample of CSO, there was no interaction between the oleogel and hydrogel while an interaction between the air and oleogel occurred. For samples containing hydrogels, the higher A values indicated the increased interactions between the two phases, and according to Figure 5D, this was related to the amount of oleogel present, resulting in samples with enhanced structural stability and mechanical properties [8], due to the interpenetration of the oleogel and hydrogel network. These properties could be related to the ability of the inks to maintain layer-by-layer adhesion after printing [8].

### 2.4. Inks’ Microstructure Analysis

**Figure 6:**
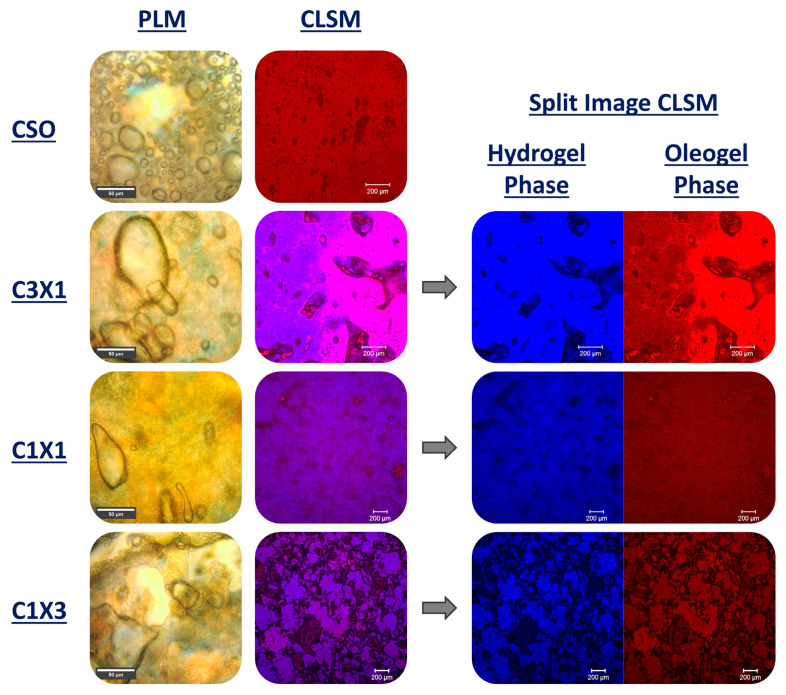
Polarized Light Microscopy (scale bar set at 50 μm) and Confocal Laser Scanning Microscopy (scale bar set at 200 μm) micrographs, along with CLSM split images (scale bar set at 200 μm) showing the two different phases (oleogel and hydrogel) present in the samples.

PLM micrographs of different ink formulations were used to interpret the distribution of the air and crystalline area (oleogel phase) within the bulk of the inks. As presented in Figure 6, the CSO sample exhibited a relatively uniform distribution of air bubbles of various sizes within its bulk. Most of the air bubbles were round shaped, indicating that there were no intense disproportionation phenomena (air bubbles merging or volume changing following the mixing or printing process). Additionally, there was a visible bright area (no crystallinity) that was indicative of a mild phase separation, demonstrated by the formation of oil islets within the ink. The microstructures of C3X1 and C1X1 samples showed distanced and elongated larger air bubbles, suggesting the presence of disproportionation phenomena. The homogeneity of the crystalline phase was also observed in the CLSM micrographs. There was an intermediate color presented throughout the image that could be compared to a bicontinuous structure. This observation was more obvious for the C1X1 sample for which both the conventional and split CLSM images showed the evenly distributed phases. Finally, the PLM micrograph of the C1X3 sample was indicative of more intense disproportionation phenomenon along with phase separation which was the reason for lower performance of the ink during printing (trapezoid shape due to partial collapse). CLSM images of the C1X1 and C1X3 samples revealed that the air was mostly presented in the form of cracks rather than bubbles, leading to poor mechanical properties. The microstructure of these samples could also be compared to a bicontinuous system as there was an even distribution of the two phases (hydrogel and oleogel) within the bulk of the inks.

### 2.5. Thermal Behavior during Heating

**Figure 7:**
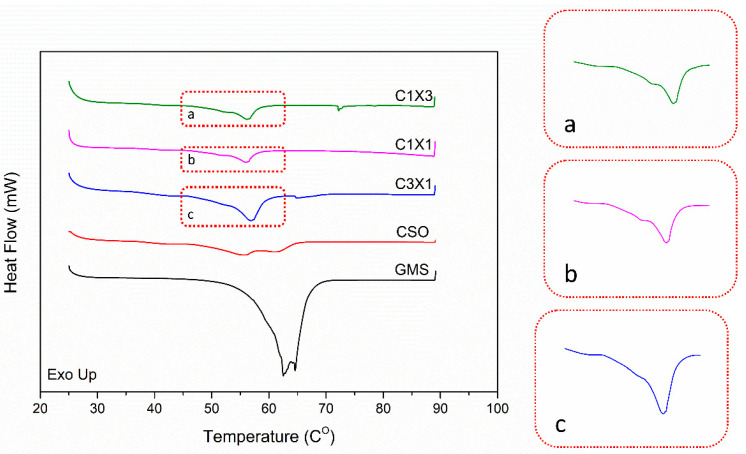
DSC thermograms of heating curves for the different ink formulations. The embedded pics a, b, and c are the zoomed areas of the thermographs that highlight the presence of the respective peaks and shoulders.

Differential Scanning Calorimetry was applied to monitor the melting behavior of the formulations during heating at temperatures that mimic the expected working temperatures of the system. DSC analysis is considered complementary to the present study as the melting behavior of the inks is indicative of the structural characteristics of the materials. Bigel formulations are expected to be mainly stabilized by the fat crystals present in the organogel phase [24]. In Figure 7, all samples exhibited a broad transition peak at around 45 °C. Only the thermograph of the CSO sample showed a distinct double peak, which was indicative of the melting of the inverse lamellar phase following the melting of the sub-α crystals [32]. The melting behavior of all the bigel formulations was characterized by the presence of a single peak with a distinct shoulder at lower temperatures, which was attributed to the presence of the hydrogel (Figure 7a–c). Additionally, the overall lower peak melting temperatures of the bigels, compared to the CSO sample (Table 2), could be attributed to a possible plasticizing effect of the hydrogel phase to the oleogel phase [32]. Finally, according to Table 2, the observed melting enthalpies of the samples were divided into two statistically similar groups: the CSO—C3X1 group and the C1X1—C1X3 group. The melting enthalpy of the CSO sample was indicative of the disruption of van der Waals forces developed by the crystal matrix of the oleogel phase. The value of the observed enthalpy was expected to be lower by reducing the fraction of the oleogel phase in the C3X1 sample, but it appears to be unaffected, due to the presence of interfacial phenomena that were induced by the hydrogel phase, increasing to some extent the observed enthalpy value, counteracting the expected lowering due to the lower oleogel fraction. This phenomenon was not observed for the second group of samples, where the observed melting enthalpy values were further decreased. This observation was attributed to the decrease in the participation of interfacial phenomena, as the system seems to transit to a bicontinuous state, as is observed in Figure 6. The results are indicative of the effect of the hydrogel concentration on the properties of the bigels and they provide useful insights for understanding the under-study materials. The transition to a bicontinuous state that was observed for C1X1 and C1X3 samples could possibly explain their inferior performance as the non-bicontinuous samples exhibit a distinct dispersion of the hydrogel phase within the oleogel phase that results in a more stable structure.

## 3. Conclusions

In this study, aerated bigel inks with varying hydrogel-to-oleogel mass ratios were used as printing materials for an extrusion-based additive manufacturing process (food 3D printing). The printability of the inks was found to be negatively affected by the presence of higher portions of the hydrogel phase, even though the printing of the bigels had been characterized as successful. The CSO ink samples exhibited superior printing performance compared to the bigel inks, while the C1X3 ink sample had inferior printing performance due to intense disproportionation along with phase separation phenomena. The presence of hydrogel had a strong effect on the mechanical properties of the inks, leading to a partial collapse of the printed structures and poor printability. The microstructure of the aerated inks was affected by the presence of a higher oleogel fraction, in terms of air bubble shape and distribution, and the results of this study also indicate the presence of intense disproportionation along with phase separation phenomena, especially for C1X3 ink. The same phenomena are also supported by the rheological and DSC study, rendering the CSO ink the best performing 3D-printable ink and the C1X3 ink sample the least favorable, in terms of their possible application as a host system for the delivery of cannabinoids, as the poor printing performance could lead to inadequate predictability of the dosing and subsequently to the delivery of the active ingredients. As an overall conclusion, the three-dimensional printing of cannabis seed oil oleogel and its bigel with xanthan gum hydrogel was plausible and promising for the development of personalized dosage forms for hosting and delivering cannabinoids. These systems are ready to be used alone or in combination with other printable food systems towards the development of a fully customizable process for the preparation of personalized cannabis edibles with tunable properties.

## 4. Materials and Methods

### 4.1. Materials

Cannabis seed oil was kindly donated from Hempoil^®^, Athens, Greece. Xanthan gum (G1253-Xanthan gum from *Xanthomonas campestris*) was purchased from Sigma-Aldrich (Zwijndrecht, The Netherlands). Glycerol Monostearate (40–55 (Type I, EP), GELEOL) was purchased from Gattefossé (Saint-Priest, France). All substances were used as received without any additional purification.

### 4.2. Sample Preparation

#### 4.2.1. Hydrogel and Oleogel Preparation

Xanthan gum (XG) hydrogels were prepared according to the literature, with modifications [2]. Briefly, XG powder was dispersed in distilled water (2% *w*/*w*) at room temperature, under continuous magnetic stirring for 24 h. Cannabis seed oil oleogels (CSOs) were prepared according to previously reported studies [32]. Glyceryl Monostearate (GMS) (20% *w*/*w*) was mixed with cannabis seed oil (80% *w*/*w*), under continuous magnetic stirring, and heated at 80 °C. When GMS was completely dissolved in CSO (clear solution), the system was kept for 5 additional minutes at 80 °C, and then it was cooled to room temperature at a cooling rate of ≤1 °C/min. The prepared oleogels were stored at 4 °C (overnight) before any further use.

#### 4.2.2. 3D-Printable Inks Preparation

The 3D-printable inks were prepared by mixing oleogels and hydrogels with different mass ratios (detailed formulations in Table 3). A syringe coupler method, inspired by the modified Tessari method for producing sclerosing foam [23], was applied to mix the two phases.

**Figure 8:**
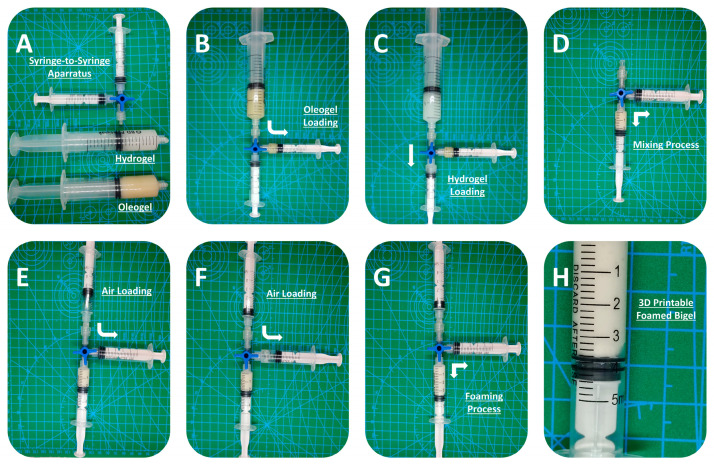
Syringe coupler mixing and foaming process. (**A**) Raw materials and mixing apparatus; (**B**) oleogel loading; (**C**) hydrogel loading; (**D**) apparatus before mixing; (**E**) apparatus after mixing (bigel formation); (**F**) air loading apparatus before foaming; (**G**) apparatus after foaming; and (**H**) foamed bigel (close-up photo).The arrows indicate movement direction.

In a typical experiment, predetermined amounts of hydrogel and oleogel were transferred to two separated 5 mL syringes that were connected to a three-way tap syringe connector (Figure 8). Following the secure lock of the syringes, the plungers were pressed back and forth (20 times or 10 cycles) to ensure thorough mixing. Afterwards, the mixture was further mixed with air using the same apparatus. For this process, one of the two syringes connected to the three-way tap connector was filled with air and subsequently mixed with the previously prepared mixture by pressing the plungers back and forth (20 times or 10 cycles). The prepared bigel foam was transferred to a new 5 mL syringe and used as a 3D-printing ink. The foamability and printability of the formulations were evaluated immediately after the air-mixing process. All samples were mixed with air at a final concentration of 20% *v*/*v*, as a benchmark concentration that was less than the higher possible amount of the incorporated air (according to Figure 1B), ensuring the accurate, precise, and reproducible incorporation of the same amount of air in all samples.

### 4.3. Overrun Measurements

The quantification of the samples’ overrun was performed to determine the overall amount of air that was capable of being incorporated within the bulk of the bigels. Overrun was determined by applying the following formula [24]:

*Overrun* (%) = 100 × (V_1_ − V_0_)/V_0_, (1)

where V_1_ is the volume of the foam after mixing the air with the bigels, while V_0_ is the initial volume of the bigel before mixing with the air.

### 4.4. Rheological Studies

Rheological measurements of the ink formulations were conducted using an Anton Paar MCR 92 (Anton Paar GmbH, Graz, Austria) strain rate-controlled rheometer equipped with a Peltier module. A 25 mm diameter parallel-plate geometry with a 1 mm gap was selected for all rheological experiments [8,32]. To evaluate the rheological properties of the samples during the extrusion process (during printing), the shear-thinning properties were evaluated and the Ostwald-de Waele power law model (Equation (2)) was applied to determine the correlation between apparent viscosity and shear rate [8,27]:

(2) $$(\eta =K\cdot {\dot{\gamma }}^{\left(n-1\right)}),$$

where *η*, $\dot{\gamma }$, *K*, and *n* are the apparent viscosity, shear rate, consistency coefficient, and power law index, respectively.

To evaluate the recovery ability of the 3D-printable inks, a thixotropy experiment with three different shear rates (1/s, 100/s, and 11/s) was carried out. A frequency sweep test was performed at the range of 10 to 100 rad/s within the linear viscoelastic range (LVR) of 0.01, and the weak gel model (Equation (3)) was applied, according to the literature [8,28]:

G* = (G′^2^ + G″^2^)^0.5^ = A⋅ω^1/z^, (3)

where z represents the interaction number of the bigels, and A represents the interaction strength.

### 4.5. Printability Evaluation

#### 4.5.1. Food 3D Printing Process

A conventional 3D printer (Ender 3 S1Pro 3D printer, Creality, Shenzhen, China) equipped with a syringe extrusion print head module suitable for food extrusion (LuckyBot Food Extruder, Wiibox Group Limited, Wan Chai, HongKong) was used to print the different specimens. The nozzle diameter and the layer height were 1 mm, while the printing speed was 10 mm/s.

#### 4.5.2. Printing Quality Evaluation

To evaluate the printing quality, different model shapes were printed, including 1D printing (1 mm × 20 mm single-filament/line printing), 2D printing (2, 5, and 10 mm × 20 mm single-layer printing), and 3D printing (5 mm × 10 mm × 20 mm rectangular 3D model). All layers were of the same thickness, and no additional walls were used. A concentric infill pattern (100% infill) was selected as a typical infill pattern for food 3D printing. Room temperature (25 °C) was selected as the print head and print-bed temperature. The overall printability was quantitively evaluated based on the single-line and single-layer printing performance (compared to the initial CAD model dimensions), while the 3D-printed specimens were evaluated qualitatively by comparing the 3D-scanned specimens (CR-Scan Lizard 3D scanner, Creality, Shenzhen, China) to the CAD model.

### 4.6. Confocal Laser Scanning Microscopy Observation (CLSM)

The inks’ structure was observed with an inverted Zeiss LSM 700 confocal microscope (Carl Zeiss, CZ Microscopy GmbH, Jena, Germany) in optical mode with a 20× lens. Before the examination, 10 µL of 0.1 mg mL^−1^ Nile Red and 10 µL of 0.1 mg mL^−1^ Nile Blue were added into the oil and aqueous phase, respectively [32,33]. A small amount of each ink was placed on a glass slide and was covered with a coverslip before imaging.

### 4.7. Polarized Light Microscopy Observation (PLM)

The microstructure of the prepared inks was captured using an Olympus BX 41 polarized light microscope (Olympus Corporation, Tokyo, Japan). All measurements were performed at 25 °C. The ink samples were placed on a microscope glass slide and covered by a coverslip to form a thin uniform film [32].

### 4.8. Differential Scanning Calorimetry (DSC)

Thermal analyses were performed by using a TA Instruments temperature-modulated DSC (TA Q2000). The instrument was calibrated with the indium standard for the heat flow and temperature, while heat capacity was evaluated using the sapphire standard. A nitrogen gas flow of 50 mL/min was purged into the DSC cell for all measurements. A total of 5 mg of sample was weighted and placed into hermetically sealed aluminum pans and heated from 25 to 90 °C at a heating rate of 10 °C/min. An empty DSC aluminum pan was used as a reference sample. Peak melting temperature (Tp_m_) and apparent melting enthalpy (ΔHm, J/g) of the different samples were obtained from the endothermic peaks of the thermograms [32].

### 4.9. Statistical Analysis

All measurements were performed in triplicates and mean average values were presented. Student’s *t*-test (Microsoft^®^ Excel^®^, Microsoft 365 (version 2205 Build 16.0.15225.20028), Redmond, WA, USA) was used for the analysis of the acquired data. The significance level was set at *p* < 0.05.

## Figures and Tables

**Table 1:** Consistency index (K) and flow index (*n*) of the different ink formulations.

Sample Name	*K* (10^5^ Pa*s^n^)	*n*	R^2^
CSO	215.1 ± 16.6 ^a^	−0.22 ± 0.02 ^a^	0.99
C3X1	206.2 ± 10.6 ^a^	−0.29 ± 0.01 ^a^	0.99
C1X1	100.9 ± 1.6 ^b^	0.14 ± 0.09 ^b^	0.99
C1X3	63.7 ± 3.2 ^c^	0.13 ± 0.07 ^b^	1

Different letters in the same column state significant differences (*p* < 0.05).

**Table 2:** Peak melting temperatures *T_m_* and melting enthalpies ΔHm of bigel inks with different formulations.

Sample Name	*T_m onset_* (°C)	*T_m_*_1_ (°C)	*T_m_*_2_ (°C)	*ΔH_m total_* (J/g)
CSO	45.2 ± 1.2	55.5 ± 1.6	61.2 ± 0.9	18.6 ± 3.9 ^a^
C3X1	45.4 ± 1.3	51.4 ± 1.1 *	56.9± 1.3	23.0 ± 2.9 ^a^
C1X1	45.2 ± 0.9	51.1 ± 0.8 *	56.1 ± 1.7	9.9 ± 2.6 ^b^
C1X3	45.1 ± 1.0	51.7 ± 1.2 *	56.3 ± 1.7	11.2 ± 2.7 ^b^

* Temperature refers to a peak shoulder; different letters in the same column state significant differences (*p* < 0.05).

**Table 3:** Different formulations of bigel inks.

Sample Name	Cannabis Seed Oil Oleogel (% *w*/*w*)	Xanthan Gum Hydrogel (% *w*/*w*)
CSO	100	0
C3X1	75	25
C1X1	50	50
C1X3	25	75

## Data Availability

The original contributions presented in the study are included in the article, further inquiries can be directed to the corresponding author.
